# Multiscale ecological boundaries and microbial community coalescence in host-associated microbiota

**DOI:** 10.1128/msphere.00058-25

**Published:** 2025-11-28

**Authors:** Benjamin T. Camper, Sharon A. Bewick

**Affiliations:** 1 Department of Biological Sciences, Clemson University 2545, Clemson, South Carolina, USA; Nanjing University of Chinese Medicine, Nanjing, Jiangsu, China

**Keywords:** ecotone, ecocline, host-associated microbiota, ecological boundary, hybrid, microbial community coalescence

## Abstract

Ecological boundaries between different environments are important for generating and maintaining biodiversity and for understanding community assembly. Classic examples include temperature gradients between upland and lowland habitats and estuaries (brackish) between fresh- and saltwater. While numerous studies have examined community assembly of free-living organisms across ecological boundaries, few studies have considered community assembly of host-associated (HA) organisms, including HA microbiota, across similar ecological boundaries. This is likely because it is unclear what constitutes an ecological boundary for organisms that reside on a host. We identify hybrid hosts (i.e., hosts derived from breeding events between two different genetic lineages) as ecological boundaries for HA organisms. More specifically, we argue that the intermediate genetic compositions of hybrid organisms, often accompanied by traits that are either intermediate to or else combinations of progenitor traits, create transition zones between the environments experienced by microbes on or in progenitor host lineages. This, in turn, paves the way for microbial community coalescence (i.e., mixing of microbial communities) that is directly analogous to community assembly in classic ecological boundaries. Further, because many hybrid hosts reside along ecological boundaries themselves, hybrid microbiota often experience simultaneous boundaries of both their hosts and their hosts’ environment—an underexplored phenomenon that we term a “multiscale ecological boundary.” By introducing ecological boundaries into HA microbiota literature, our goals are to further understanding of HA microbiota assembly and to propose a framework for studying the effects of ecological boundaries at multiple scales.

**IMPORTANCE:**

Boundaries between environments provide important insight into how ecological communities are structured across broader landscapes. Of particular interest is how communities assemble within the transition zone constituting the boundary (i.e., where the transition in environmental variables occurs) and whether transitions in community composition parallel transitions in environmental variables. While ecological boundaries have a long history in classic ecology, similar concepts have recently emerged in microbiota literature. Currently, however, most studies of microbial ecological boundaries focus on environmental microbiota, rather than host-associated (HA) microbiota. This is likely because it is unclear what constitutes an ecological boundary in HA microbiota systems. We propose hybrid hosts as an HA analog for environmental ecological boundaries. Specifically, we outline how different types of hybrid hosts serve as models for different types of ecological boundaries. We then outline how the ecological boundary framework can be used to interpret HA microbial community coalescence (i.e., mixing) across host species. Finally, we suggest that many hybrid hosts reside within the transition zones of larger scale ecological boundaries. When this happens, hybrid hosts can be used to examine a novel phenomenon that we term a “multiscale ecological boundary.”

## OPINION/HYPOTHESIS

Ecological landscapes typically comprise multiple regions with different environmental characteristics. At the contact zones between these regions, there is often a space segment (the “transition zone”) over which environmental variables change (i.e., an environmental gradient). Termed an ecological boundary, these transition zones have long been of interest for their unique effects on biodiversity. This includes their propensity to be colonized by novel combinations of species, to select for rare alleles and/or phenotypes, and to support higher species richness (1). Because of their unique characteristics, ecological boundaries make excellent models for studying the ecological and evolutionary processes contributing to generation and maintenance of biodiversity, including speciation, species interactions, and community assembly (2). Two of the most-studied ecological boundaries are ecotones and ecoclines. Ecotones occur when the abiotic gradient is discontinuous between two regions with different environmental characteristics (e.g., lake shoreline between terrestrial and aquatic habitat). By contrast, ecoclines occur when the transition zone is a more gradual environmental gradient (e.g., elevational gradient spanning a mountain slope). Both types of ecological boundaries have been described across a broad range of spatial scales and appear to affect community assembly of widely disparate taxa; thus, they reflect widespread drivers of patterns in species distributions, community composition, and gene flow (3).

While macrobial systems have been the focus of most research on ecological boundaries, recent studies have begun to examine similar phenomena in microbial systems. This includes explicit studies of how environmental gradients drive microbial community gradients across landscape-level ecotones and ecoclines. It also includes the related field (4, 5) of microbial community coalescence, a new research area focused on understanding the results of mixing (i.e., “coalescence”) microbial communities through processes like direct physical combination or blending of environmental variables (i.e., environmental or abiotic coalescence). Though not formally recognized, the latter is, in essence, the definition of a classic ecological boundary. Despite recent interest in ecological boundaries within microbial literature, most existing work has focused on environmental microbiota. Indeed, to the extent that host-associated (HA) microbiota have been considered at all (6), the emphasis has been on understanding the boundary between the host and its environment (7) or between different host individuals. This is more akin to studying variation in community composition between regions with different environments or between different replicates of the same environment (e.g., different ponds) than it is to studying variation across ecological boundaries between two environments (e.g., the pond to terrestrial transition). The current emphasis of HA microbial systems on pure environments, rather than boundaries, points to one of the main challenges of studying ecological boundaries in HA microbiota. HA organisms experience their environment as a combination of both the host on which they reside (i.e., host level) and the environment in which their host resides (i.e., landscape level). This complicates the definition of an ecological boundary for HA organisms. Further, whereas landscape-level ecological boundaries are relatively easy to identify, it is less clear what constitutes a host-level ecological boundary.

Given the emerging paradigm that hosts create the environment for their associated microbiota, we identify different host lineages as the conceptual HA analogs of environmental classes occupied by free-living organisms (8). By extension, hybrid hosts (i.e., organisms derived from breeding events between two different genetic [progenitor] lineages) can be considered the conceptual HA analogs of ecological boundaries. By viewing hybrid hosts as host-level ecological boundaries, we can extend foundational concepts of landscape ecology to HA microbial community coalescence. This not only allows for testing classic ecological hypotheses but also raises new questions about community assembly processes in HA microbiota. In particular, because hybrid hosts often reside along landscape-level ecological boundaries themselves (9), the HA microbiota of many hybrid organisms simultaneously experience the effects of ecological boundaries at both the host and landscape level—a phenomenon that we term a “multiscale ecological boundary.” Although multiscale processes have been increasingly recognized for their contributions to HA microbial community assembly (8), multiscale ecological boundaries have not been discussed. We argue that the HA microbiota of hybrid hosts provide a unique window into these unexplored multiscale processes that may have important implications for understanding HA microbiota and ecological communities more broadly.

### Ecotones

Ecotones occur in regions with sharp boundaries between different types of environments. In this scenario, the boundary is characterized by discrete environmental attributes that are distinct from adjacent environments on either side. An example is the high salt marsh that lies between marsh plain environment and upland environment along the California coast (10). In ecotones, community composition generally changes abruptly between the ecotone and the adjacent environments; however, within the ecotone, environmental variables and, as a result, community composition tend to be relatively homogeneous or, at the very least, do not exhibit the same gradual variation between adjacent environments that defines an ecocline (see below) (1). In some cases, the uniqueness of ecotone communities emerges through the appearance of new species (ecotone specialists) (1–3). More commonly, however, the uniqueness emerges from the novel convergence of species that are found in one or other of the environments adjacent to the ecotone. Ecotones are of interest to ecologists because they house unique species and species combinations. Ecotones are of interest to evolutionary biologists because they introduce different and often strong selective pressures on their inhabitants, even to the point of inducing speciation (11). Although the unique effects of ecotones have been documented in free-living microbial communities (4), their effects on HA microbiota remain largely unknown.

### Ecoclines

Typically induced by a gradient in one or more environmental variables (e.g., temperature or pH), ecoclines are gradual boundaries between different types of environments. Widespread examples are the salinity gradients that form along many river estuaries connecting inland freshwater environments to the ocean (3). As compared with ecotones, communities within ecoclines typically exhibit a more continuous transition in composition, smoothly interpolating between the communities found in environments at either end. Like ecotones, however, ecoclines often support unique combinations of species that independently inhabit the environments at their borders. Notably, because ecoclines are heterogeneous, communities harbored within ecoclines often exhibit compositional structuring along the gradient (i.e., community gradient) (1, 3). Also, for this reason, the selective pressures associated with ecoclines are not as uniformly extreme as those in ecotones (12). Thus, ecoclines tend to be more permeable. This may be why they tend to harbor higher biodiversity than ecotones relative to the environments at their respective borders (13). However, for this same reason, gene flow may be more persistent across ecoclines, and thus, ecoclines may be less likely to induce speciation. Like ecotones, the unique effects of ecoclines have been documented in free-living microbial communities (5). Again, however, the effects of ecoclines on HA microbiota are poorly known.

### Hybrid hosts as ecotones and ecoclines

Hybrid hosts offer a useful lens through which to study ecological boundaries and how ecological boundaries impact microbial community coalescence/microbial community gradients in HA microbial systems. Just as the ecotones and ecoclines of free-living organisms are the result of mixing environmental characteristics between two different landscape-level environments, hybrid hosts are the result of mixing traits between two different host-level environments (i.e., host lineages) (14). Importantly, many of the traits that are mixed in hybrid offspring are traits that could conceivably impact the microbial environment and, by extension, HA microbiota assembly (e.g., diet [15] or immune system function [16]). Thus, hybrid organisms can be thought of as ecological boundaries between their progenitor species.

**Fig 1:**
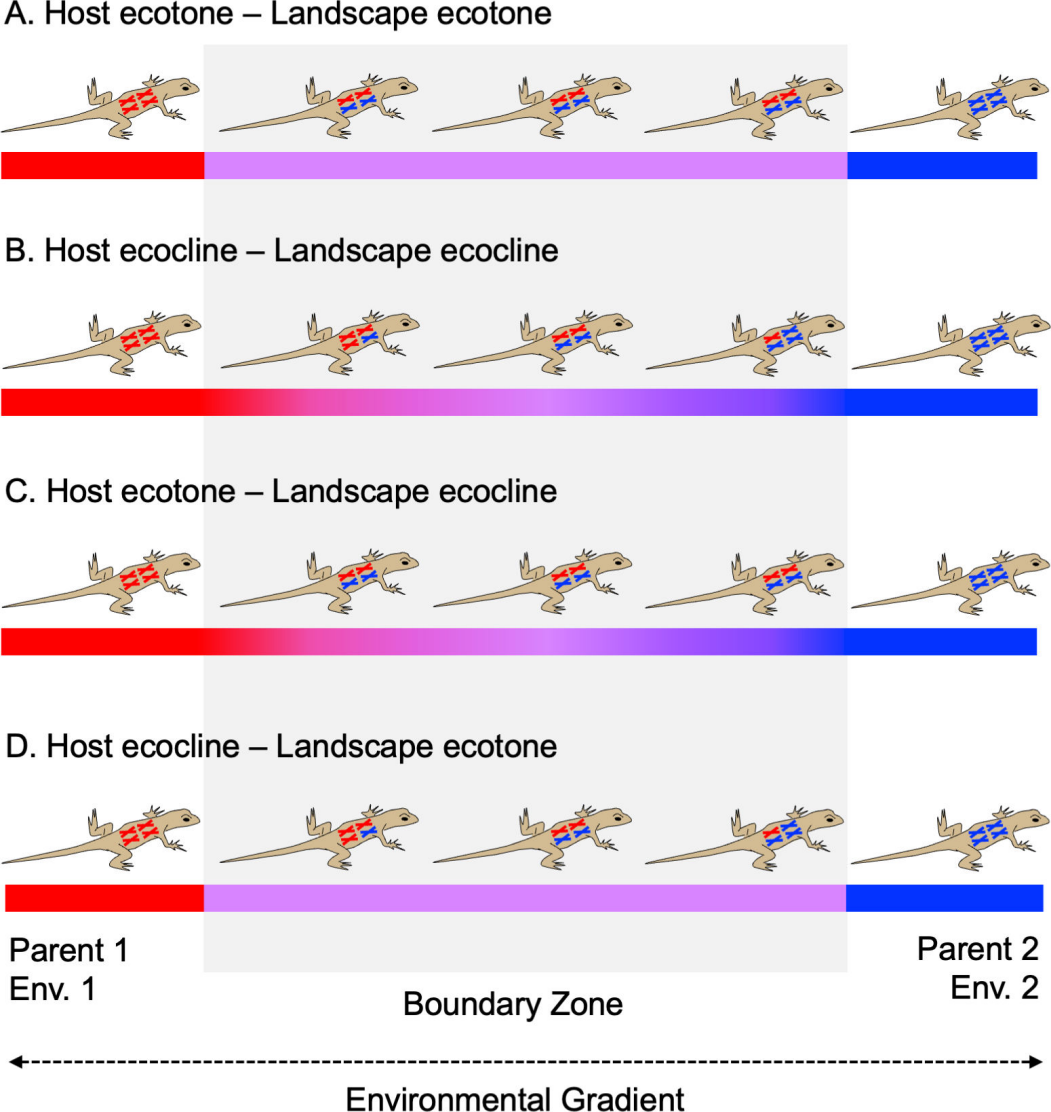
Schematic illustrating possible combinations of multiscale ecological boundaries (i.e., host environmental gradients) outlined in this manuscript. Host-level (i.e., lizard) boundaries are depicted as varying genomic content of the host (colored chromosomes), with all hosts that harbor both blue and red chromosomes representing hybrids. In this example, host-level ecotones possess no genomic variation (**A, C**), whereas host-level ecoclines display a spectrum of genomic variation between each adjacent host-level environment (**B, D**). Landscape-level boundaries are depicted using colored lines; the two environments adjacent to the boundary are shown in red and blue, and ecotones/ecoclines are shown as shades of purple. Whereas there is an abrupt transition from red/blue to purple in landscape-level ecotones (**A, D**), the change is more gradual in landscape-level ecoclines (**B, C**). Note that ecological boundaries are defined based on the environmental conditions within the transition zone. Here we depict differing host-level environmental conditions using the genetic composition of the host. This, however, is meant to represent myriad different genetically determined traits (body size, metabolite production, MHCs) that may create or contribute to the host-level environment.

Some hybrid hosts resemble ecotones while others resemble ecoclines (see Fig. 1). Hybrid hosts resembling ecotones emerge when hybrids are characterized by abrupt and discrete differences from their progenitors while being relatively homogeneous amongst themselves. This can occur in systems where hybrids are constrained from backcrossing with progenitor species. The result is uniform populations of first-generation (F_1_) hybrids with intermediate genetic composition that underlies discrete phenotypic or ecological differences from progenitors (9). Broadly speaking, backcrossing may be prevented by either endogenous factors, such as hybrid sterility, or exogenous factors, such as occupation of a novel niche that prevents reproductive contact with progenitors (14). In either case, hybrid hosts end up similar to one another, but at the same time, very distinct from both progenitors. Mules are a classic example of endogenously derived host-level ecotones (hybrid sterility) (17), while *Larus occidentalis × L. glaucescens* gulls provide an example of an exogenously derived host-level ecotone (niche differentiation) (18).

In contrast to ecotones, hybrid hosts resembling ecoclines emerge when hybrids exhibit variable genetic composition and/or trait differentiation that spans the spectrum between their two progenitor lineages (9). This can occur in systems where hybrid hosts freely, or at least frequently interbreed with one another and backcross with progenitors, resulting in variable degrees of admixture into both progenitor lineages, as well as a range of genetically and/or phenotypically intermediate individuals. A good example of a host-level ecocline is the hybrid zone between *Picea glauca* and *P. engelmannii* in western Canada (19). These hybrid spruce trees can be found spanning the entire range of genotypes intermediate to the two progenitor species and exhibit several growth and survival traits that differ from progenitors as well.

**Fig 2:**
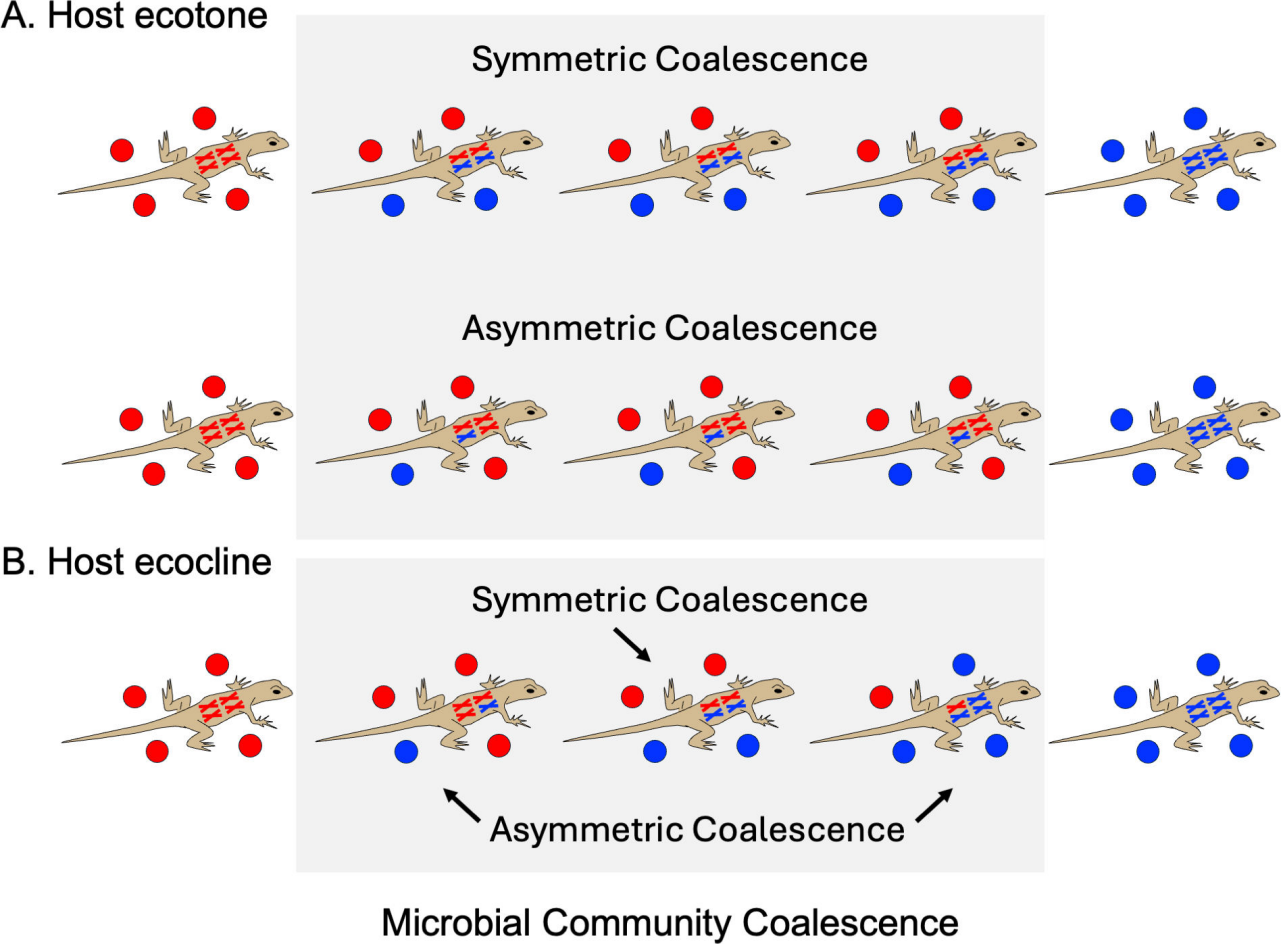
Schematic illustrating possible effects of host-scale ecotones (**A**) and ecoclines (**B**) on microbial community coalescence/microbial community gradients. Host-level boundaries are depicted as varying genomic content of the host (see Fig. 1 for reference), while HA microbial communities are depicted as circles surrounding each lizard. For both ecotones and ecoclines, progenitor microbiota can be represented in a 50:50 ratio (symmetric community coalescence) or in any other ratio (asymmetric community coalescence). Whether and how HA microbiota coalesce across host hybrid complexes and how this depends on the larger landscape in which the system is embedded (see Fig. 1) remain open questions.

Just as classic ecotones and ecoclines can provide insight into community assembly of free-living organisms, hybrid hosts can provide insight into community assembly of HA organisms (see Fig. 2 for several community outcomes). More specifically, in hybrid hosts, the mixing of two distinct host genomes and their regulatory networks creates opportunity for hybrid novelty via mechanisms such as *cis* and *trans* regulatory changes (20), epigenomic changes (21), and transcriptomic shock (22). The altered host environment can then drive microbial community coalescence that is directly analogous to the community assembly of free-living organisms within classic ecological boundaries. Like landscape-level boundaries, which can be defined by a range of different environmental variables (e.g., temperature, precipitation, soil type), different aspects of the host environment may underlie different host-level ecological boundaries. In some systems, for instance, microbes may respond to changes in host morphological features (23). In other systems, it could be differences in the host immune system (e.g., major histocompatibility complexes) (24, 25), host physiology (e.g., metabolite production) (26) or even host ecology (e.g., diet preference) (27).

One potential difference between hybrid organisms and classic ecological boundaries is that environmental variables within classic ecological boundaries are usually intermediate to or else combinations of the environmental variables of adjacent environmental classes. While this is also true for the genetics of hybrid organisms, host genetics do not directly determine the environment experienced by HA microbes. Rather, host genetics exert their effects via host traits. In this respect, it is important to note that, beyond genetics, many hybrids exhibit traits that are either intermediate to or else combinations of progenitor traits (28). In a study of synthetic hybrid plants between *Argyranthemum broussonetii* and *A. frutescens*, for example, hybrids were intermediate for all nine measured traits (29). While intermediacy is not true for all traits in all hybrid systems, when it is true, hybrid organisms serve as direct HA analogs for the ecological boundaries of free-living organisms. In cases where important hybrid traits are not intermediate to or combinations of progenitor traits, hybrid hosts may still represent HA ecological boundaries; however, these boundaries may not have an analog at the landscape scale.

Another difference between hybrid organisms and classic ecological boundaries is that most classic ecological boundaries are spatially adjacent to the environmental classes that they connect. This facilitates spatially structured dispersal of organisms from pure environmental classes into the transition zone. While there are plenty of hybrid systems where hybrids persist in spatial transition zones (e.g., tension zones) (30), even to the point of forming spatially structured genetic and/or phenotypic gradients between pure progenitor lineages, this is not always the case. In some systems, hybrids of differing genetic composition and/or phenotype mix freely (i.e., host-host contact that enables inter-individual microbial dispersal), and in other systems, these hybrids even mix with progenitor lineages themselves. Further, there are systems where hybrid organisms reside in geographic ranges and/or environmental classes fully discontiguous from progenitor lineages. In systems where hosts are not spatially structured according to genetic status and/or host traits, hybrid organisms can still be viewed as ecological boundaries; however, community assembly within these boundaries will not be driven by spatially structured dispersal from pure (progenitor) environmental classes in the same way that it is in landscape-level ecological boundaries. This does not mean that microbiota assembly in these HA systems will not mirror community assembly in certain landscape-level ecological boundaries. However, the equivalent boundaries at the landscape scale will be those governed by environmental filtering rather than dispersal limitation (e.g., plants with wind-dispersed seeds).

### Multiscale ecological boundaries

While hybridizing systems are geographically and phylogenetically widespread (see Shurtliff [31], Barton and Hewitt [32] and Abbott [9] for reviews of dozens of hybrid systems constituting as host-level ecological boundaries), numerous hybrid zones are restricted to or otherwise reside in regions with landscape-level ecological boundaries. In fact, in many cases, it is these landscape-level boundaries that putatively underlie the emergence and/or maintenance of hybrid organisms in the first place (9). Ecotones and ecoclines are, for example, common locations of hybrid zones because they coincide with regions of reproductive contact between host lineages that are largely restricted to adjacent environmental classes. Additionally, some hybrids exhibit superior fitness to progenitors in ecotones (30) or ecoclines (9); thus, these ecological boundaries can provide competitive refugia for hybrids, facilitating the coexistence of hybrids and their progenitors within landscapes. When hybrid host populations occur within or across landscape-level ecotones or ecoclines, the HA microbiota on the hybrid hosts experience ecological boundaries at both the host and landscape levels. How host- and landscape-level boundaries interact to determine HA microbiota assembly remains unknown and may provide insight into the largely unexplored phenomenon of multiscale ecological boundaries.

Notably, in regions with both host- and landscape-level boundaries, the boundaries that exist at each scale may or may not be of the same type (ecotone vs ecocline, see Fig. 1) and may or may not be spatially coincident. A host-level ecotone may, for example, span a landscape-level ecocline (Fig. 1C), while a host-level ecocline may span a landscape-level ecotone (Fig. 1D). Likewise, hybrid organisms may be restricted to the landscape-level ecotone/ecocline, or they may extend into the environments on either side, while progenitor species may be restricted to adjacent environments or may disperse into the ecotone/ecocline. In many cases, however, the combination of dispersal limitation and selective pressure within the hybrid zone supports emergence of relatively spatially coincident host- and landscape-level boundaries of the same type (Fig. 1A and B; i.e., multiscale ecotones or multiscale ecoclines).

### Classic patterns and new questions

Extending the concepts of ecological boundaries to hybrid hosts enables us to examine a range of classic landscape ecology patterns in HA microbiota. This can help to identify mechanisms governing HA microbiota assembly and put patterns of microbial community coalescence into a broader ecological context. Additionally, it allows for testing classic ecological hypotheses in new systems. For instance, are HA communities in host-level ecoclines (i.e., hybrid microbiota) more diverse than HA communities in adjacent host-level environments (i.e., progenitor microbiota)? (13) If so, is this because of convergence of microbial taxa into the host-level ecoclines from adjacent host-level environments? To what extent do host-level ecological boundaries (i.e., hybrid microbiota) harbor unique microbial taxa (2), and does this differ depending on whether you consider host-level ecotones versus host-level ecoclines? How do microbial communities vary across host-level ecoclines, and to what extent is this due to turnover versus nestedness?

Beyond classic patterns, the multiscale nature of HA ecological boundaries raises new questions. For instance, do host- and landscape-level ecological boundaries have additive or antagonistic effects on HA microbiota diversity? While additive effects may be more likely, interesting interactions between scales may emerge in scenarios where landscape- and the host-level environmental gradients have opposing or otherwise interactive effects on certain microbial taxa. This could impact the symmetry of resulting microbial community coalescence (6) relative to either the host (see Fig. 2) or landscape gradient and could make the outcomes of interacting scales difficult to predict. A related multiscale question is how spatial variation in communities differs when host- and landscape-level ecological boundaries are of the same versus different types (i.e., ecotone vs. ecocline). Again, this could yield interesting and nonintuitive outcomes on spatial variation in HA microbiota. Microbial communities could, for example, preferentially assemble in response to one type of boundary, regardless of scale (e.g., HA communities always assemble in response to the ecotone regardless of whether the ecotone occurs at the host or landscape level). Alternatively, microbial communities could preferentially assemble in response to one scale regardless of type (e.g., HA communities always assemble in response to the host scale regardless of whether it is an ecotone or ecocline). A third interesting question is whether unique microbial taxa are more common in host- or landscape-level ecotones. One might predict that unique taxa are most common in regions where host- and landscape-level ecotones coincide, highlighting the potential for interactions of ecological boundaries across scales to introduce novelty into host-microbe systems (i.e., holobionts). Notably, these questions are novel not only to HA microbiota literature, but to community ecology more broadly, and could be asked in any system where multiscale ecological boundaries occur (e.g., soil microbial communities structured along a landscape ecocline and across soil strata).

While the coincident nature of host- and landscape-level ecological boundaries makes strong multiscale interactions possible, this can also confound efforts to separate the independent effects at each scale. One obvious solution is to house hybrid complexes under identical conditions in captivity. This would allow testing for the independent effects of host-level ecological boundaries in the absence of landscape effects. Alternatively, some hybrid systems are distributed, such that it is possible to find both locations where landscape-level boundaries coincide with host-level boundaries, as well as locations where boundaries at each scale occur independently. In Supplemental Information I, we discuss two example model systems—one for ecotones and one for ecoclines—with some of these characteristics.

### Summary

Ecological boundaries and their effects on microbial community coalescence remain poorly explored in HA microbiota. This is likely a result of challenges in defining what constitutes an ecological boundary for HA organisms. By introducing hybrid hosts as HA analogs to ecological boundaries in free-living organisms, we extend foundational concepts from landscape ecology into the HA microbiota lexicon. By doing so, we introduce a testable framework for exploring microbial community coalescence in host systems and align this with classic ecological literature. Beyond introducing new systems for testing classic hypotheses and providing context for understanding HA microbiota assembly and microbial community coalescence, our framework highlights new multiscale ecological concepts. Importantly, these multiscale concepts are not unique to HA microbiota or even microbial systems more broadly. Rather, theories developed to explain HA microbiota assembly on hybrid hosts could be extended to a range of additional macrobial and microbial systems, including both HA and free-living organisms wherever multiscale ecological boundaries exist.

## SUPPLEMENTAL MATERIAL

Supplemental Information msphere.00058-25-s0001.docx Example model systems for host-associated ecotones and ecoclines.

ASM does not own the copyrights to Supplemental Material that may be linked to, or accessed through, an article. The authors have granted ASM a non-exclusive, world-wide license to publish the Supplemental Material files. Please contact the corresponding author directly for reuse.
