# Supplementary material for: Multiscale ecological boundaries and microbial community coalescence in host-associated microbiota
Source: mSphere. 2025 Nov 28;10(12):e00058-25. doi: 10.1128/msphere.00058-25 (PMC12724255; doi:10.1128/msphere.00058-25)
Supplement: Supplemental Information — Example model systems for host-associated ecotones and ecoclines. [file msphere.00058-25-s0001.docx]

**Multiscale ecological boundaries and microbial community coalescence in host-associated microbiota**

**Supplemental Information I: Model Systems**

Whiptail lizards as models for multiscale ecotones: Hybrid clonal lineages of *Aspidoscelis* lizards (formerly *Cnemidophorus*) are widely distributed across the southwestern United States and adjacent Mexico. Arising from hybridization between different progenitor species, these hybrid lineages maintain themselves, genetically isolated from both progenitors, by cloning the F_1_ hybrid state *in perpetuum.* This results in relatively homogeneous populations of hybrid lizards with fully intermediate genotypes to their progenitors.^1^ As such, clonal *Aspidoscelis* lizards are excellent models of host-level ecotones. Further, many clonal *Aspidoscelis* co-occur extensively with their progenitors, existing sympatrically and even syntopically.^2^ Some also prefer ecotonal environments, though most are broadly distributed, including in environments inhabited by one or both progenitors.^3^ Regions of syntopy (hybrids and progenitors) allow for testing of host-level ecotones in the absence of landscape-level ecotones. Regions where clones span both the ecotone and adjacent environments on either side allow for testing of landscape-level ecotones in the absence of host-level ecotones. Finally, regions where clones inhabit ecotones that connect progenitor environments allow for testing of coincident host- and landscape-level ecotones. Importantly, this means that it is possible to use the HA microbiota of clonal *Aspidoscelis* lizards to examine both classic and multiscale patterns across ecotones *in situ* and without manipulating animals. While *Aspidoscelis* clones are a great example of a homogeneous host-level ecotone (i.e., all animals are nearly identical), this does mean that the ecotone is represented by a single genotype. Thus, HA microbiota differences in the ecotone could potentially emerge due to idiosyncrasies of the specific genotype involved. The numerous *Aspidoscelis* clonal lineages, including multiple origins of hybridization by the same progenitors, however, can be used to largely mitigate this particular challenge.

Fire-bellied toads as models for multiscale ecoclines: *Bombina bombina* and *B. variegata* hybridize along mountain-lowland ecoclines in Central and Eastern Europe. Importantly, there are at least 11 geographically isolated hybrid zones that lie along otherwise similar elevational gradients. In many of these hybrid zones, there is extensive back-crossing of hybrids with progenitors, resulting in well-covered genomic clines, with animals that span the full range of genetic relatedness between progenitors.^4^ Thus, hybrid *Bombina* are excellent models of host-level ecoclines. Importantly, however, different hybrid zones exhibit different levels of admixture and introgression, as well as differences in the steepness of the genomic clines. Further, despite all of these hybrid zones occurring along elevational gradients, altitude is a poor predictor of *B. bombina* versus *B. variegata* gene frequencies across hybrid zones.^5^ Thus, by comparing HA microbiota across different hybrid zones, it is possible to disentangle the independent and combined effects of host- and landscape-level ecoclines *in situ* and without manipulating animals.

**References**

1. Reeder TW, Cole CJ, Dessauer, Herbert C. Phylogenetic Relationships of Whiptail Lizards of the Genus *Cnemidophorus* (Squamata: Teiidae): A Test of Monophyly, Reevaluation of Karyotypic Evolution, and Review of Hybrid Origins. *Am Mus Novit*. Published online 2002:1-61. doi:10.1206/0003
2. Camper BT, Kanes AS, Laughlin ZT, Manuel RT, Bewick SA. Transgressive hybrids as hopeful holobionts. *Microbiome*. 2025;13(19):1-26. doi:10.1186/s40168-024-01994-8
3. Wright JW, Lowe CH. Weeds, Polyploids, Parthenogenesis, and the Geographical and Ecological Distribution of All-Female Species of *Cnemidophorus*. *Copeia*. 1968;1968(1):128-138.
4. Dufresnes C, Suchan T, Smirnov NA, Denoël M, Rosanov JM, Litvinchuk SN. Revisiting a speciation classic: Comparative analyses support sharp but leaky transitions between Bombina toads. *J Biogeogr*. 2021;48(3):548-560. doi:10.1111/jbi.14018
5. Gollmann G, Roth P, Hödl W. Hybridization between the fire-bellied toads *Bombina bombina* and *Bombina variegata* in the karst regions of Slovakia and Hungary: morphological and allozyme evidence. *J Evol Biol*. 1988;1:3-14.
